# *Polygonum orientale* L. Alleviates Myocardial Ischemia-Induced Injury via Activation of MAPK/ERK Signaling Pathway

**DOI:** 10.3390/molecules28093687

**Published:** 2023-04-24

**Authors:** Changli Fu, Mingjin Wang, Yuan Lu, Jie Pan, Yueting Li, Yongjun Li, Yonglin Wang, Aimin Wang, Yong Huang, Jia Sun, Chunhua Liu

**Affiliations:** 1 State Key Laboratory of Functions and Applications of Medicinal Plants, Engineering Research Center for the Development and Application of Ethnic Medicine and TCM (Ministry of Education), Guizhou Medical University, Guiyang 550004, China; 2 School of Pharmacy, Guizhou Medical University, Guiyang 550004, China; 3 Guizhou Provincial Key Laboratory of Pharmaceutics, Guizhou Medical University, Guiyang 550004, China

**Keywords:** *Polygonum orientale* L., myocardial ischemia, MAPK signaling pathway

## Abstract

Although *Polygonum orientale* L. (PO) has a beneficial effect on treatment of myocardial ischemia (MI), its mechanism remains unclear. This study aimed to explore the pharmacological mechanism of PO against MI through MAPK signaling pathways. Firstly, the therapeutic effect of PO was evaluated for treatment of MI mice. Using Western blot and immunohistochemistry, the influence of PO on MAPK signaling pathways and cell apoptosis was investigated. Subsequently, one key pathway (ERK) of MAPK signaling pathways was screened out, on which PO posed the most obvious impact. Finally, an inhibitor of ERK1/2 was utilized to further verify the regulatory effect of PO on the MAPK/ERK signaling pathway. It was found that PO could reduce the elevation of the ST segment; injury of heart tissue; the activity of LDH, CK, NOS, cNOS and iNOS and the levels of NO, BNP, TNF-α and IL-6. It is notable that PO could significantly modulate the protein content of p-ERK/ERK in mice suffering from MI but hardly had an effect on p-JNK/JNK and p-p38/p38. Additionally, the expressions of bax, caspase3 and caspase9 were inhibited in heart tissue in the PO-treated group. To evaluate whether ERK1/2 inhibitor (PD98059) could block the effect of PO on treatment of MI, both PO and PD98059 were given to mice with MI. It was discovered that the inhibitor indeed could significantly reverse the regulatory effects of PO on the above indicators, indicating that PO could regulate p-ERK/ERK. This study provides experimental evidence that PO extenuates MI injury, cardiomyocyte apoptosis and inflammation by activating the MAPK/ERK signaling pathway.

## 1. Introduction

Myocardial ischemia (MI), one of the common cardiovascular diseases with high mortality and morbidity worldwide, results from coronary atherosclerosis or blockage of myocardial blood supply and oxygen supply [1,2]. Included among the complex mechanism of MI are mainly energy metabolism disorder, oxygen free radical injury, oxidative stress, apoptosis and inflammation [3]. At present, the conventional treatment is the use of drug eluting stents, coronary artery bypass graft surgery, anti-thrombosis and so on [4]. Drug therapy is the most acceptable treatment, with low damage to patients, so it is necessary to develop more drugs for the treatment of MI.

China has a long and distinguished history of traditional Chinese medicine (TCM) for treatment or prevention of diseases, dating back thousands of years [5]. *Polygonum orientale* L. (PO), a Chinese medicine derived from the family Polygonum, has an obvious effect on treating such diseases as coronary heart disease, chest tightness and shortness of breath [6]. In previous studies, our team has systematically studied its chemical composition, extraction process, quality control, pharmacological activity and pharmacokinetics during the treatment of acute MI [7,8,9], but there is a scarcity of information on its mechanism. Intriguingly, it was found that with the method of network pharmacology in the preliminary study, main targets of components of PO were involved in the mitogen-activated protein kinase (MAPK) signaling pathway, suggesting MAPK signaling pathways may play important roles in PO against MI. MAPK signaling pathways could regulate many biological processes, including the cell cycle, differentiation, apoptosis, stress, inflammation and protein biosynthesis [10], also playing an important role in the pathogenesis of cardiac and vascular disease [11]. Extracellular signal-regulated kinase 1 and 2 (ERK1/2), c-jun N-terminal kinase (JNK) and p38 are the members of MAPK signaling pathways [10]. ERK1/2 could regulate cell differentiation and proliferation, promoting cell survive and conferring tissue protection [12,13]. It has been reported that p38 and JNK accelerate apoptosis and injury during MI, but ERK1/2 has anti-apoptotic and anti-inflammatory effects [14]. Moreover, ERK1/2, JNK and p38 has an indispensable role in cardiac hypertrophy, cardiac remodeling after myocardial infarction, atherosclerosis and vascular restenosis [11,15,16]. Other studies claimed that with the mechanism of the increasing number of drugs investigated, regulating the MAPK signaling pathway is a key approach for treatment or prevention of MI [17,18,19]. Hence, the study was designed to investigate whether the cardioprotective effect of PO on MI is mediated by MAPK signaling pathways in MI mice using molecular biological experimental techniques, together with an inhibitor.

## 2. Results

### 2.1. Chemical Profile of PO Extract

**Figure 1:**
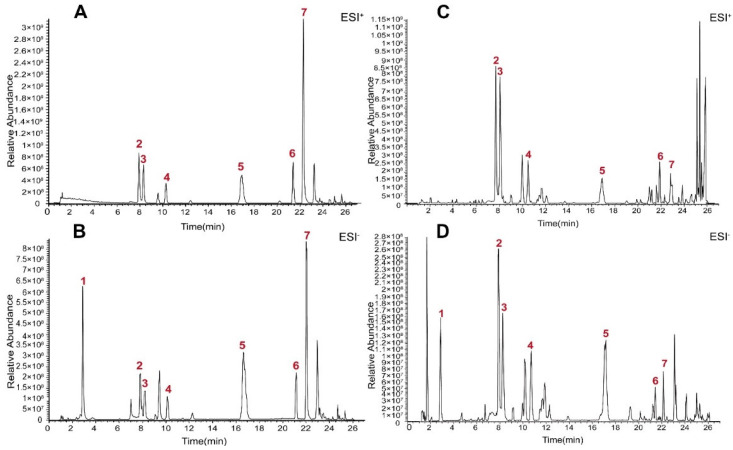
BPC of PO extract and mixed reference solution using UHPLC–Q-Exactive Orbitrap Plus HRMS. (**A**) BPC of mixed reference solution in positive ion modes. (**B**) BPC of mixed reference solution in negative ion modes. (**C**) BPC of PO extract in positive ion modes. (**D**) BPC of PO extract in negative ion modes. (1: protocatechuic acid, 2: isoorientin, 3: orientin, 4: vitexin, 5: quercetin, 6: *N*-*p*-*trans*-coumaroyltyramine, 7: *N*-*trans*-feruloyltyramine).

The base peak chromatograms (BPCs) of PO extract and standard solutions are displayed in Figure 1. The main chemical compounds included protocatechuic acid, isoorientin, orientin, vitexin, quercetin, *N*-*trans*-feruloyltyramine and *N*-*p*-*trans*-coumaroyltyramine in PO extract.

### 2.2. Effect of PO on Electrocardiogram (ECG) in MI Mice

**Figure 2:**
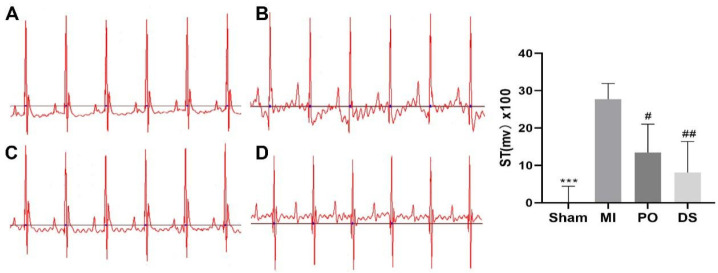
The effect of PO on ST segment. (**A**) Sham group. (**B**) MI group. (**C**) PO group. (**D**) DS group. (Mean ± SD, *n* = 6, *** *p* < 0.001 vs. MI group; ^#^ *p* < 0.05, ^##^ *p* < 0.01 vs. MI group).

As showed in Figure 2, the ST segment of mice in the MI group was significantly elevated compared with the sham group (*p* < 0.001), indicating that the MI model was established successfully. However, the ST segment was significantly decreased in PO and Danshen dripping pills (DS) groups (*p* < 0.05 or *p <* 0.01), showing that PO could significantly improve the elevation of the ST segment caused by MI.

### 2.3. Effect of PO on MI Injury and Inflammatory Response

**Figure 3:**
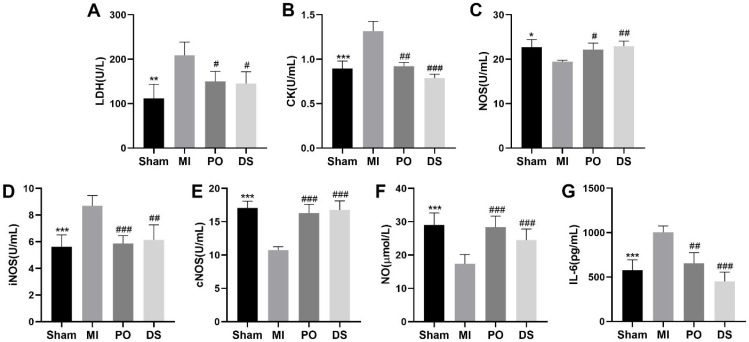
PO regulates MI injury, coronary artery contraction and relaxation and inflammatory in MI mice. (**A**) LDH. (**B**) CK. (**C**) NOS. (**D**) iNOS. (**E**) cNOS. (**F**) NO. (**G**) IL-6. (Mean ± SD, *n* = 6, * *p* < 0.05, ** *p* < 0.01, *** *p* < 0.001 vs. MI group; ^#^ *p* < 0.05, ^##^ *p* < 0.01, ^###^ *p* < 0.001 vs. MI group).

Compared with the sham group, the activities of LDH and CK in serum were markedly enhanced in the MI group (*p* < 0.01 or *p* < 0.001), while pretreatment with PO and DS significantly inhibited the activities of LDH and CK (*p* < 0.05, *p* < 0.01 or *p* < 0.001) (Figure 3A,B), suggesting that PO exerts beneficial cardioprotection against MI injury. Additionally, as Figure 3C–G showed, the activities of nitric oxide synthase (NOS) and constitutive NOS (cNOS) and the level of nitric oxide (NO) were significantly lower than the sham group (*p* < 0.05 or *p* < 0.001), while the activity of inducible NOS (iNOS) and the expression of IL-6 were higher (*p <* 0.001). Nevertheless, both PO and DS were able to observably ameliorate these indicators (*p <* 0.05, *p <* 0.01 or *p* < 0.001). These results reveal that PO could improve the function of regulating coronary artery contraction and relaxation and decrease inflammatory reaction.

### 2.4. Effects of PO on Apoptosis in MI Mice

**Figure 4:**
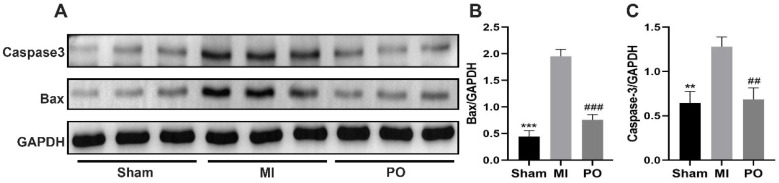
PO reduces apoptosis resulting from MI. (**A**) The protein expressions of bax and caspase3. (**B**,**C**) Quantitative analysis of bax and caspase3. (Mean ± SD, *n* = 3, ** *p* < 0.01, *** *p* < 0.001 vs. MI group; ^##^ *p <* 0.001, ^###^ *p* < 0.001 vs. MI group).

To determine whether PO for treatment of MI inhibited apoptosis, the protein levels of bax and caspase3 were examined in heart tissue of mice. There was a significant increase caused by MI in bax and caspase3 levels compared to the sham group (*p <* 0.01 or *p* < 0.001), but there appeared a significant decrease in bax and caspase 3 relative to the MI group (*p* < 0.001 or *p* < 0.001) (Figure 4). These results indicate that PO could reduce apoptosis resulting from MI.

### 2.5. Effects of PO on MAPK Signaling Pathways in MI Mice

**Figure 5:**
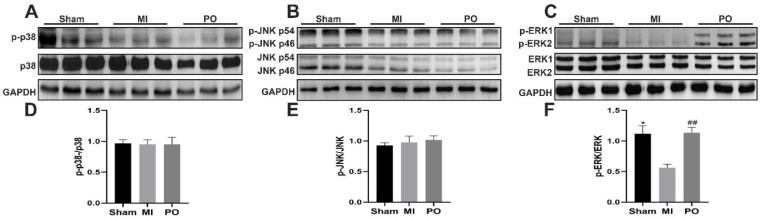
Effect of PO on MAPK signaling pathways. (**A**) Western blot bands of p38 and p-p38. (**B**) Western blot bands of JNK and p-JNK. (**C**) Western blot bands of ERK1/2 and p-ERK1/2. (**D**) Quantitative analysis of p-p38/p38. (**E**) Quantitative analysis of p-JNK/JNK. (**F**) Quantitative analysis of p-ERK1/2/ERK1/2. (Mean ± SD, *n* = 3, * *p <* 0.05 vs. MI group, ^##^ *p <* 0.01 vs. MI group).

In order to further explore whether PO affected the MAPK signaling pathways after MI. The data from Western blot tests showed that there was no significant change in the expression of p-JNK/JNK and p-p38/p38 among the sham group, MI group and PO group. However, it is worth noting that the expression of p-ERK/ERK significantly decreased in the MI group compared with the sham group (*p* < 0.05), but the expression of p-ERK/ERK significantly increased (*p <* 0.01) in mice after administration of PO (Figure 5). It is suggested that the mechanism of PO for treatment of MI may be related to the activation of the ERK1/2 signaling pathway. Subsequently, for the purpose of verifying this finding, the inhibitor (PD98059) of ERK1/2 was used to evaluate the anti-MI effect of PO.

### 2.6. Involvement of ERK Signaling Pathway in the Cardioprotective Effect of PO on MI Mice

**Figure 6:**
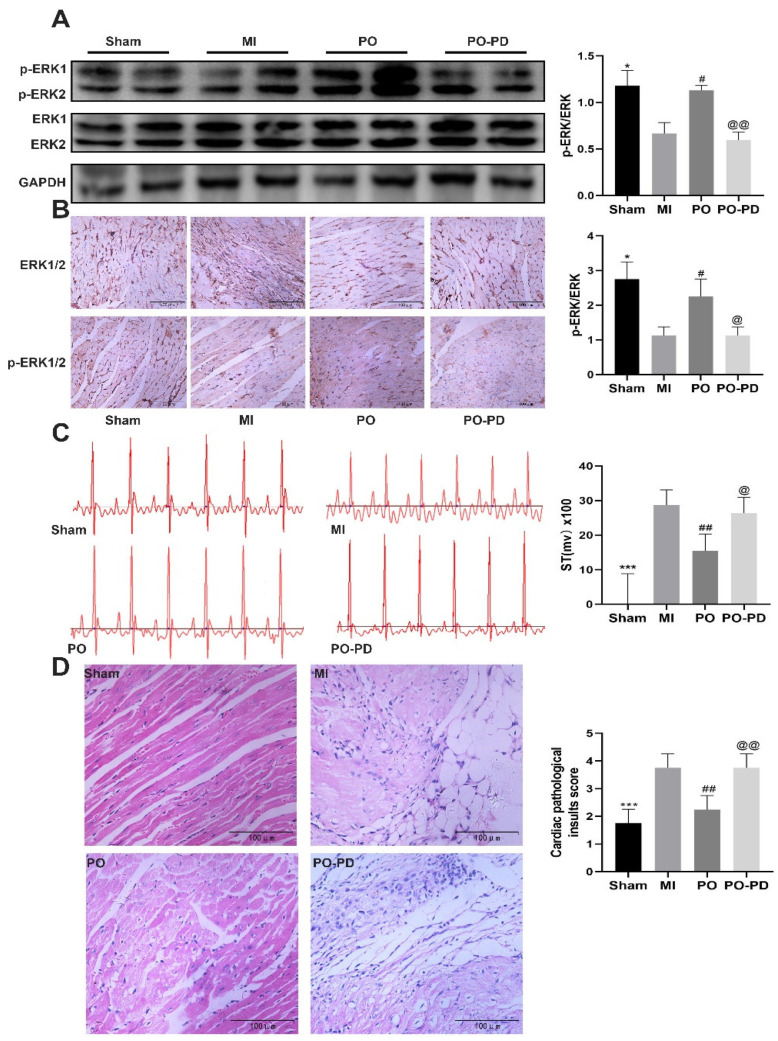

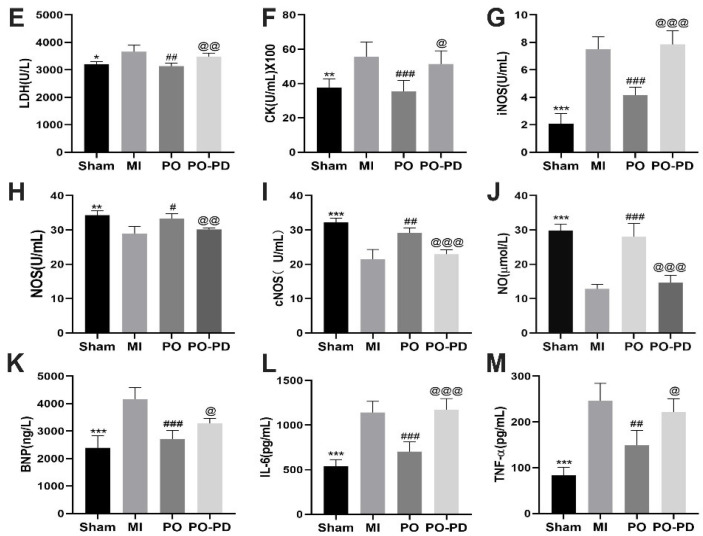
Involvement of ERK pathway in the cardioprotective effect of PO on MI mice. (**A**) Western blot bands and quantitative analysis of ERK and p-ERK1/2, *n* = 3. (**B**) Immunohistochemistry and quantitative analysis of ERK and p-ERK1/2, *n* = 4. (**C**) PO decreases the ST segment, *n* = 6. (**D**) PO improves myocardial pathological injury, *n* = 4. (**E**–**J**) The activities of LDH, CK, iNOS, NOS and cNOS in serum, *n* = 6. (**K**–**M**) The level of BNP, IL-6 and TNF-α in serum, *n* = 6. (Mean ± SD, *p < 0.05, ** *p* < 0.01, *** *p <* 0.001 vs. MI group; ^#^ *p* < 0.05, ^##^ *p* < 0.01, ^###^ *p* < 0.001 vs. MI group; ^@^ *p* < 0.05, ^@@^ *p* < 0.01, ^@@@^ *p* < 0.001 vs. PO group).

The expressions of ERK1/2 and p-ERK1/2 were detected using Western blot and immunohistochemistry. Compared with the sham group, the level of p-ERK decreased in the MI group (*p* < 0.05), while its level in the PO group was significantly higher than that in the MI group (*p* < 0.05). Nevertheless, the p-ERK level in the PO–PD (PO–PD98059) group was significantly lower than in the PO group (*p* < 0.05), indicating that the effect of PO on activation of ERK1/2 was blocked by PD98059 in MI mice (Figure 6A,B). As shown in Figure 6D, compared with the sham group, there was obvious myocardial degeneration, necrosis, vacuolation, fibroblast proliferation and inflammatory cell infiltration in the model group, and the histopathological injury was reduced after PO treatment. However, PD98059 could inhibit the effect of PO in MI. In addition, compared with the sham group, the ST segment and the activities of LDH, CK and iNOS in the MI group were significantly increased (Figure 6C,E,F, *p <* 0.05, *p <* 0.01 or *p <* 0.001). In the MI group, the activities of NOS, cNOS and NO level significantly decreased (Figure 6H–J, *p <* 0.01, *p <* 0.001), and the contents of BNP, IL-6 and TNF-α significantly increased (Figure 6K–M, *p <* 0.001) relative to the sham group. In the PO-treated group, however, PO significantly adjusted the changes of the ST segment, myocardial degeneration and necrosis as well as LDH, CK, iNOS, NOS, cNOS, NO, BNP, IL-6 and TNF-α (*p* < 0.05, *p* < 0.01 or *p* < 0.001). Compared to the PO group, the improvements conferred by PO disappeared in the PO–PD group, suggesting that PO indeed could regulate the ERK1/2 signaling pathway to decrease MI injury, cardiomyocyte apoptosis and inflammation.

### 2.7. Effect of PO on Apoptosis-Related Proteins through Activating ERK Signaling Pathway

**Figure 7:**
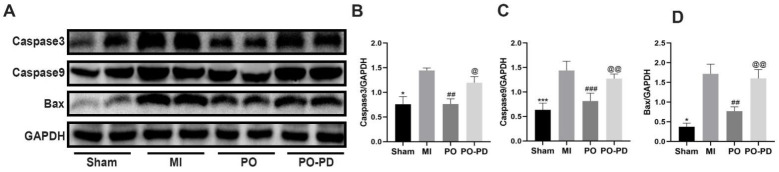
PO reduces the apoptosis related proteins through activating ERK signaling pathway. (**A**) Western blot bands of casepase3, caspase9 and bax. (**B**–**D**) Quantitative analysis of casepase3, caspase9 and bax. (Mean ± SD, *n* = 3, * *p* < 0.05, *** *p* < 0.001 vs. MI group; ^##^ *p* < 0.01, ^###^ *p* < 0.05 vs. MI group; ^@^ *p* < 0.05, ^@@^ *p <* 0.01 vs. PO group).

The ERK1/2 signaling pathway is involved in the process of cell growth and differentiation in MI [20]. On the basis of the results above, PO could boost the level of p-ERK1/2, so we further explored the effect of activating the ERK1/2 signaling pathway on apoptosis through detecting the expression of apoptosis-related proteins such as casepase3, caspase9 and bax using Western blot. It was shown (Figure 7) that the expressions of casepase3, caspase9 and bax in the MI group significantly increased (*p* < 0.05 or *p* < 0.001), while PO could make them significantly lower (*p* < 0.001 or *p* < 0.01) in the PO group. However, the expressions of casepase3, caspase9 and bax in the PO–PD group were significantly higher (*p* < 0.05 or *p <* 0.01) than that in the PO group, indicating that PO indeed could activate the ERK1/2 signaling pathway to reduce the apoptosis induced by MI.

## 3. Discussion

PO, a Chinese medicine with good medicinal value derived from family Polygonum, is commonly used in rheumatoid arthritis, CAD, stomachache and other diseases [8]. In previous studies it was discovered that the MAPK signaling pathway likely plays a regulatory role in the process of PO for treatment of MI using network pharmacology. Furthermore, the MAPK signaling pathway is also involved in multiple biological processes [10]. Therefore, this study was designed to investigate whether PO alleviates myocardial ischemia-induced injury via MAPK signaling pathways.

In this study, a range of indicators were tested to investigate the efficacy of PO on improvement of MI, including the change of the electrocardiogram; the activities of LDH, CK, NOS, iNOS and cNOS and the levels of NO and apoptotic proteins in mice. The effect of PO on the key proteins p38, JNK and ERK1/2 in the MAPK signaling pathway was analyzed by Western blot. Interestingly, the results showed that there were no significant changes in the regulation of p38 and JNK during the process of PO treating MI, but PO could significantly activate the ERK1/2 signaling pathway. Furthermore, it was confirmed that this activation of ERK1/2 could be impeded by the inhibitor PD98059. There was evidence showing that PO might increase the activities of NOS and cNOS and the level of NO; decrease the activities of iNOS, LDH and CK and the level of BNP; inhibit the content of inflammation-related factors IL-6 and TNF-α and reduce the expression of apoptosis-related proteins. Accordingly, this influence could be blocked by PD98059, suggesting that the ERK1/2 signaling pathway was involved in the process during which PO exerted therapeutic effect on MI.

Extracellular signal-regulated kinases (ERKs), specifically ERK1 and ERK2, are key enzymes that transmit signals from surface receptors to the nucleus [12]. The ERK1/2 activation cascade, also known as the Ras/Raf/MEK/ERK pathway, regulates a variety of kinase activities involved in cell proliferation and differentiation, cytoskeleton construction, apoptosis and other biological responses [20]. In recent years, an increasing number of studies have reported that activating the ERK1/2 signaling pathway plays a protective role in MI, myocardial ischemia-reperfusion injury and diabetic heart disease [18,21,22]. In our present study, through the pretreatment of inhibitor PD98059, the regulatory effect of PO on ERK1/2 protein was inhibited, showing that activating the ERK1/2 signaling pathway was a vital molecular pathway for PO against myocardial injury.

It is widely acknowledged that MI can give rise to an increase in oxygen free radicals destroying the balance between oxidants and antioxidants, resulting in myocardial injury and the release of such myocardial enzymes as LDH and CK [23,24]. BNP, with the highest content in the heart, is recognized as an important index for the diagnosis of heart failure, frequently distributing in the brain, heart and lung [25]. It had been reported that when the heart muscle suffers from ischemia and hypoxia, the local contraction of the heart is limited, thus pulling the normal cardiomyocytes around the ischemic tissue, which leads to the synthesis and release of BNP [26]. The more BNP is synthesized and released, the more severe the myocardial ischemic injury. In this study, PO can reduce the level of BNP, indicating that PO can improve myocardial contractile function and injury degree.

It has been reported that activation of the ERK signaling pathway can reduce the inflammatory response in MI injury [27]. Inflammation is one of the key pathogeneses of myocardial injury. According to the literature [28], IL-6 is a biomarker of inflammation used to evaluate the severity and prognosis of coronary heart disease. TNF-α level was closely related to the progression of myocardial ischemic injury, myocardial ischemia/reperfusion, myocardial remodeling and heart failure [29]. In our study, with the levels of IL-6 and TNF-α measured, it was found that PO could decrease the level of IL-6 and TNF-α in MI by activating the ERK1/2 signaling pathway. Additionally, the inflammatory infiltration of cardiomyocytes was decreased, which was similar to the results from the investigation conducted by Wang M. [30].

NO, a main vasodilator in the body and an important endogenous myocardial protective factor, plays an important role in regulating the cardiovascular system [31]. Endogenous NO is a free radical gas catalyzed by NOS in vivo [32]. NOS can be divided into two types: cNOS and iNOS. cNOS includes endothelial NOS and neuronal NOS. It has been reported that under pathological conditions, overproduction of NO by an iNOS may be detrimental to contractile function, while NO produced by cNOS is likely to be an important regulator of cardiac contractile function, promoting cardiac systole [33,34]. The final effect of NO and NOS on myocardial ischemia depends on the combined effect of NO produced by iNOS and cNOS, respectively. Although the trend of NO and iNOS is different in our experiment, it is consistent with the trend of total NOS and cNOS. Therefore, the reason for the different trend of NO and iNOS may be that the production of NO is mainly influenced by cNOS in myocardial ischemia. It was revealed that dexmedetomidine protects the heart from ischemia/reperfusion injury through an endothelial cNOS/NO-dependent mechanism [35]. iNOS can lead to local and systemic inflammation and cardiac remodeling in ischemic heart failure [36]. In patients attacked by decompensated chronic heart failure, there was a significant linear correlation between iNOS activity and plasma BNP level [37]. For instance, resveratrol prevents isoproterenol-induced myocardial infarction in rats via the VEGF-B/AMPK/eNOS/NO signaling pathway [38]. Through the experiment using isolated perfused mouse hearts, combined with ERK1/2 inhibitor U1026, it was found that the anti-apoptosis and cardioprotective mechanism of NO in ischemia-reperfusion heart is associated with the activation of ERK [12]. Similarly, we also found that PO can activate ERK1/2 to increase NOS, NO and cNOS to dilate blood vessels and boost myocardial blood supply.

Of great importance is apoptosis among mechanisms of myocardial ischemic injury [39]. In MI injury, increased expression of ERK1/2 protein can inhibit the expression of bax, caspase3 and caspase9 [40]. Selective inhibition of proteolytic function of caspase-3 may be an attractive method to reduce or reverse heart failure [41]. It has been reported that *Polygonum orientale* flower extract can significantly increase the protein phosphorylation level of ERK1/2 protein in hypoxia-reoxygenation injury of H9c2 cardiomyocytes and decrease caspase3 and bax to inhibit cardiomyocyte apoptosis [42,43]. The results of this study showed that the expression of caspase3, caspase9 and bax increased during MI, while PO could reduce their expression by activating the ERK1/2 signaling pathway, indicating that the mechanism of PO for treatment of MI is also linked to anti-apoptosis.

This study preliminarily confirmed that PO can reduce myocardial pathological injury; decrease the levels of IL-6, BNP and TNF-α in serum; increase the activities of NOS and cNOS and the level of NO and decrease the levels of iNOS, caspase3, caspase9 and bax by activating the ERK1/2 signaling pathway in MI mice.

## 4. Materials and Methods

### 4.1. Animals

Adult KM mice (25–30 g) were procured from the animal center of Guizhou Medical University (Guiyang, China, certificate NO SCXK 2018-0001). They were housed adaptively for 1 week in an animal house at a suitable temperature (18–25 °C) and humidity (50–70%) and had free access to water and food. The study protocol was approved by the Animal Ethics Committee of Guizhou Medical University Animal Center (Guiyang, Guizhou, China, certificate no. 1801209).

### 4.2. Chemicals and Reagents

Enzyme-linked immunosorbent assay (ELISA) kits for determination of IL-6 (KE10007) and TNF-α (KE10002) were provided by Proteintech Group, Inc. (Wuhan, Hubei, China). BNP(H166), NOS(A014-1), LDH(A020-2), CK (A032-1) and NO (A013-2-1) test kits were purchased from Jiancheng Bioengineering institute (Nanjing, China). PD98059 (lot no. MKCN2664) was obtained from Sigma Aldrich (St. Louis, MO, USA). Anti-caspase3 (9662S), anti-caspase9 (95047) and bax (27727) were purchased from Cell Signaling Technology (Shanghai, China). Anti-JNK (ab179461), anti-phosphorylation JNK (ab124956), anti-p38 (ab170099), anti-phosphorylation p38 (ab195049), anti-ERK1/2 (ab184699), anti-phosphorylation ERK1/2 (ab214036) and anti-GAPDH (ab181603) were obtained from Abcam (Shanghai, China). The BCA and enhanced chemiluminescence kits were bought from Dalian Meilun Biotechnology Co., Ltd. (Dalian, China). Tribromoethanol (C11707118) and tert-pentyl (F2004116) alcohol were supplied by Shanghai Macklin Biochemical Co., Ltd. and Shanghai Aladdin Bio-Chem Technology Co., Ltd., respectively. Protocatechuic acid (lot no. AF6121206), *N*-*trans*-feruloyltyramine (AF20060301), orientin (lot no. AF9052413), isoorientin (lot no. AF20051551), vitexin (lot no. AF8111891) and *N*-*p*-*trans*-coumaroyltyramine (lot no. AF20060304) were obtained from Chengdu Alfa Biotechnology Co., Ltd. (Chengdu, China). Quercitrin (lot no. wkq18041101) was provided by Sichuan Weikeqi Biological Technology Co., Ltd. (Chengdu, China), the purity of which was more than 98%.

### 4.3. Preparation of PO Extract

The extract of PO was prepared through referring to the previous experimental method [44]. Briefly, the medicinal parts of PO (10 kg) were extracted with 10-fold water for 1 h for 3 times and concentrated into a solution of 1 g/mL. Subsequently, 95% ethanol was added and stirred, making the solution contain ethanol of 65% for 12 h. The solution was filtered to collect filtrate, which was concentrated to recycle ethanol in vacuum. Then, the filtrate was extracted with 5 L of water-saturated n-butanol 4 times, and the n-butanol extract was collected and evaporated to acquire residue using a vacuum evaporator. The residue dissolved in 80% ethanol was transferred onto a polyamide column and eluted with 80% ethanol. The ethanol eluate was collected and dried with a vacuum direr to obtain the PO extract (1.95%, yield). In this study, the concentrations of the main components (protocatechuic acid, isoorientin, orientin, vitexin, kaempferol-3-O-β-D-glucoside, quercetin, *N*-*trans*-feruloyltyramine and *N*-*p*-*trans*-coumaroyltyramine) of PO were 29.49, 115.26, 151.58, 24.09, 2.03, 79.79, 5.98 and 2.64 mg/g, respectively.

PO extract (about 10 mg) was weighed and dissolved in 10 mL of 50% aqueous methanol, and then the solution was filtered through a 0.22 μm membrane, the filtrate of which was collected for identification of chemical profiles of PO using UHPLC–Q-Exactive Orbitrap Plus HRMS.

### 4.4. Preparation of Standard Solutions

The reference compounds were accurately weighed and dissolved in 10 mL methanol to obtain the stock solution. Mixed reference solution (2–10 μg/mL) was prepared by diluting each stock solution with 50% methanol.

### 4.5. UHPLC–Q-Exactive Orbitrap Plus HRMS Analysis Conditions

The analysis was performed on a Vanquish horizon UHPLC coupled with Q-Exactive Plus HRMS Spectrometer (Thermo Fisher, Waltham, MA, USA). The Q-Exactive Plus HRMS Spectrometer equipped with an electrospray ionization (HESI) source was employed for MS in both positive and negative modes. The MS conditions were as follows: spray voltage 3.5 kV for positive ion mode and 2.5 kV for negative ion mode, capillary temperature 320 °C, ion source sheath gas flow rate 35 arb, aux gas flow rate 10 arb, probe heater temperature 350 °C and the stepped NCE was set to 20, 40 and 60 V. The scan ranges of a full MS scan with a resolution of 70,000 were set to m/z 100–1500, and dd-MS2 at a resolution of 17,500 was recorded from 100 to 1500 m/z.

Chromatographic separation was performed on a Hypersil gold column (2.1 × 100 mm, 1.9 μm) at 40 °C with mobile phase A (water containing 0.1% formic acid, *v*/*v*) and B (acetonitrile), at the flow rate of 0.3 mL/min and with the injection volume of 2 μL. The gradient elution procedure was as follows: 6% B from 0 to 0.5 min, 6–8% B from 0.5 to 2 min, 8–13.5% B from 2 to 5 min, 13.5% B from 5 to 21 min, 13.5–30% B from 21 to 35 min, 30–80% B from 35 to 38 min and 80–95% B from 38 to 39 min.

### 4.6. Establishment of MI Model

Mice were anaesthetized with intraperitoneal injection of tribromoethanol (350 mg/kg) with tert-pentyl alcohol. According to a method reported in references to establish the MI model [45], briefly, mice were connected to HX-300S animal ventilator (Taimeng Software co. Ltd., Chengdu, China) after anaesthetization. The chest cavity of mice was cut open to expose the heart, and then the left anterior descending coronary artery was ligated with a 6-0 suture to block the myocardial blood supply. After alcohol disinfection incision, the gas in the chest cavity was squeezed out, and the cut was quickly sutured. The sham group suffered the same operation, but the coronary artery was not ligated.

### 4.7. Experimental Protocol

The experimental protocol was divided into two parts. The first was designed to explore the effect of PO on MI in mice and the regulation of the MAPK signaling pathway. Mice were divided into four groups (*n* = 6), namely a sham group, myocardial ischemia group (MI), PO group (PO, crude drug of PO extract 4 g/kg) and Danshen dripping pills (DS, 12.27 mg/kg) group. Danshen dripping pills are clinically used to treat coronary artery disease [46], so they were selected as the experimental control group. In the current study, the design of the administration dose of PO was based on our previous research [47], and the DS group was based on clinical dose. PO group and DS group mice were given PO and DS in 0.5% sodium carboxymethylcellulose (CMC-Na) with oral administration for 14 consecutive days, while other mice were given the same volume of 0.5% CMC-Na with the same means. The following indicators were assessed as the activity of LDH, CK, NOS, cNOS and iNOS in serum: the levels of NO and IL-6 in serum and protein expression of p38, p-p38, ERK1/2, p-ERK1/2, JNK, p-JNK, bax, caspase3 and caspase9 in heart tissue.

The second was intended to investigate and verify the role of ERK1/2 signaling in the process during which PO exerted a protective effect on MI. The mice were divided into four groups (*n* = 6): the sham group, myocardial ischemia group (MI), PO group (PO) and PO–PD98059 group (PO–PD). The mice in the PO group were orally administered PO extract (4 g/kg). For the PO–PD group, before administering PO extract for 30 min, mice were intraperitoneally injected with PD98059 (1 mg/kg) based on Reference [48]. The mice in the sham group and MI group were given the same volume of 0.05% CMC-Na. All mice were treated for 14 consecutive days. The degree of pathological injury in myocardial tissue was evaluated using H&E staining. The activities of LDH, CK, NOS, iNOS and cNOS and the levels of NO, BNP, IL-6 and TNF-α were determined. Finally, the expression of ERK1/2 and p-ERK1/2 was detected by Western blot and immunohistochemistry.

### 4.8. ECG

After a 14-day treatment, mice were anesthetized and placed on a small platform with ECG recording electrodes for determination of ECG.

### 4.9. Pathological Evaluation of Myocardial Tissue

The left ventricle was fixed with 4% neutral paraformaldehyde. Myocardial tissue was dehydrated using alcohol of gradient concentration, transparent with xylene, and soaked and embedded into paraffin blocks. Paraffin blocks were sliced into 4 μm-thick sections, which were dewaxed with xylene, hydrated and stained with hematoxylin–eosin. Lastly, the degree of pathological changes was observed under an optical microscope, then scored based on Rezkalla’s score standard [49]. The specific scoring criteria were as follows: normal, 0; the degree of inflammatory cell infiltration, degeneration and necrosis was <25%, 1; the degree of inflammatory cell infiltration, degeneration and necrosis was 25% to 50%, 2; the degree of inflammatory cell infiltration, degeneration and necrosis was 50% to 75%, 3; the degree of inflammatory cell infiltration, degeneration and necrosis was ≥75%, 4.

### 4.10. Detection of Myocardial Enzyme

Orbital blood was collected and centrifuged at 1000× *g* for 10 min at 4 °C to collect serum. Then, the activities of LDH, CK, NOS, cNOS and iNOS and the level of NO were determined strictly according to the requirements of the corresponding kits.

### 4.11. ELISA Assay

The concentrations of IL-6, BNP and TNF-α in serum were detected based on the protocols of commercial ELISA kits.

### 4.12. Western Blot Analysis

Heart tissue was homogenized and centrifuged (15,221× *g*, 10 min, 4 °C) to take the total protein, concentration of which was measured by a BCA kit. Firstly, the total proteins were separated using 10% SDS-PAGE, then transferred to PVDF membranes. After blocking with efficient Western blocking fluid for 15 min, the membranes were incubated with the following specific primary antibodies overnight at 4 °C: anti-p38 (1:5000), anti-p-p38 (1:1000), anti-ERK1/2 (1:5000), anti-p-ERK1/2 (1:1000), anti-JNK (1:1000), anti-p-JNK (1:5000), anti-GAPDH (1:10,000), anti-bax (1:1000), anti-caspase3 (1:1000) and anti-caspase9 (1:1000). Subsequently, these membranes were incubated with adding HRP-conjugated secondary antibodies (1:5000) for 2 h at room temperature. Lastly, the membranes were visualized using a gel imaging system with an enhanced chemiluminescence kit and quantified using Quantity One software.

### 4.13. Immunohistochemistry Analysis

The myocardial tissue was embedded in paraffin and sliced into 4 μm-thick sections. After dewaxing and hydration, the slices were incubated with 3% hydrogen peroxide for 10 min at room temperature and washed 3 times with PBS. Antigen retrieval was performed with ethylenediaminetetraacetic acid disodium salt (0.001 mol/L pH8.0). The sections were incubated overnight at 4 °C with primary antibodies such as anti-ERK1/2 (1:500), anti-p-ERK1/2 (1:480) and PBS (a negative control). Then, sections were reacted with goat anti-rabbit or mouse HRP-conjugated secondary antibodies at 37 °C for 30 min. Diaminobenzidine was used to visualize the reaction product. Finally, the sections were washed, then stained with hematoxylin and finally visualized under a microscope.

### 4.14. Statistical Analysis

Data were analyzed using GraphPad Prism software 7 and expressed as mean ±SD. One-way analysis of variance (ANOVA) was used for comparison when more than two groups were compared. *p* < 0.05 was considered statistically significant.

## 5. Conclusions

In summary, the mechanism of PO for treatment of MI may be that PO activates the MAPK/ERK signaling pathway, downregulating the expression of such apoptosis-related proteins as bax, caspase3 and caspase9; improving myocardial inflammation; increasing the NOS activity and NO level in serum to dilate blood vessels and increase the myocardial blood flow and oxygen supply.

## Data Availability

All the relevant data are provided within the paper, and data in the current study are available from the corresponding author.

## Sample Availability

These samples in the current study are available from the authors.

## Abbreviations

BPC	base peak chromatogram
CMC-Na	sodium carboxymethylcellulose
cNOS	constitutive NOS
CAD	coronary artery disease
ECG	electrocardiogram
ELISA	enzyme-linked immunosorbent assay
ERK1/2	extracellular signal-regulated kinase 1 and 2
JNK	c-jun N-terminal kinase
MAPK	mitogen-activated protein kinase
MI	myocardial ischemia
iNOS	inducible NOS
NO	nitric oxide
NOS	nitric oxide synthase
PO	*Polygonum orientale* L.
TCM	traditional Chinese medicine
